# Comparative Analyses of the Clinicopathologic Features of Short-Term and Long-Term Survivors of Patients with Pancreatic Ductal Adenocarcinoma Who Received Neoadjuvant Therapy and Pancreatoduodenectomy

**DOI:** 10.3390/cancers15123231

**Published:** 2023-06-18

**Authors:** Tom Z. Liang, Matthew H. G. Katz, Laura R. Prakash, Deyali Chatterjee, Hua Wang, Michael Kim, Ching-Wei D. Tzeng, Naruhiko Ikoma, Robert A. Wolff, Dan Zhao, Eugene J. Koay, Anirban Maitra, Suprateek Kundu, Huamin Wang

**Affiliations:** 1 Department of Pathology, The University of Texas MD Anderson Cancer Center, Houston, TX 77030, USA; tzliang@mdanderson.org (T.Z.L.); dchatterjee@mdanderson.org (D.C.); amaitra@mdanderson.org (A.M.); 2 Department of Surgical Oncology, The University of Texas MD Anderson Cancer Center, Houston, TX 77030, USA; mhgkatz@mdanderson.org (M.H.G.K.); lrprakash@mdanderson.org (L.R.P.); mkim@mdanderson.org (M.K.); cdtzeng@mdanderson.org (C.-W.D.T.); nikoma@mdanderson.org (N.I.); 3 Department of Gastrointestinal Medical Oncology, The University of Texas MD Anderson Cancer Center, Houston, TX 77030, USA; huawang@mdanderson.org (H.W.); rwolff@mdanderson.org (R.A.W.); dzhao3@mdanderson.org (D.Z.); 4 Department of Radiation Oncology, The University of Texas MD Anderson Cancer Center, Houston, TX 77030, USA; ekoay@mdanderson.org; 5 Department of Translational Molecular Pathology, The University of Texas MD Anderson Cancer Center, Houston, TX 77030, USA; 6 Department of Biostatistics, The University of Texas MD Anderson Cancer Center, Houston, TX 77030, USA; skundu2@mdanderson.org

**Keywords:** pancreatic cancer, neoadjuvant therapy, tumor response grade, tumor stage, lymph node metastasis, long-term survivors, short-term survivors

## Abstract

**Simple Summary:**

In this study, we systematically examined the clinicopathologic characteristics of 60 short-term survivors and 149 long-term survivors and compared them to 352 intermediate-term survivors of pancreatic ductal adenocarcinoma (PDAC) who received NAT and pancreatoduodenectomy. We found that lymph node stage (ypN) was an independent predictor of both short-term and long-term survivors. In addition, tumor differentiation was an independent predictor for short-term survivors, and tumor response grading and perineural invasion were independent predictors for long-term survivors. Our results may help to plan and select post-operative adjuvant therapy for patients with PDAC who received NAT and pancreatoduodenectomy based on the pathologic data.

**Abstract:**

Neoadjuvant therapy (NAT) is increasingly used to treat patients with pancreatic ductal adenocarcinoma (PDAC). Patients with PDAC often show heterogenous responses to NAT with variable clinical outcomes, and the clinicopathologic parameters associated with these variable outcomes remain unclear. In this study, we systematically examined the clinicopathologic characteristics of 60 short-term survivors (overall survival < 15 months) and 149 long-term survivors (overall survival > 60 months) and compared them to 352 intermediate-term survivors (overall survival: 15–60 months) of PDAC who received NAT and pancreatoduodenectomy. We found that the short-term survivor group was associated with male gender (*p* = 0.03), tumor resectability prior to NAT (*p* = 0.04), poorly differentiated tumor histology (*p* = 0.006), more positive lymph nodes (*p* = 0.04), higher ypN stage (*p* = 0.002), and higher positive lymph node ratio (*p* = 0.03). The long-term survivor group had smaller tumor size (*p* = 0.001), lower ypT stage (*p* = 0.001), fewer positive lymph nodes (*p* < 0.001), lower ypN stage (*p* < 0.001), lower positive lymph node ratio (*p* < 0.001), lower rate of lymphovascular invasion (*p* = 0.001) and perineural invasion (*p* < 0.001), better tumor response grading (*p* < 0.001), and less frequent recurrence/metastasis (*p* < 0.001). The ypN stage is an independent predictor of both short-term and long-term survivors by multivariate logistic regression analyses. In addition, tumor differentiation was also an independent predictor for short-term survivors, and tumor response grading and perineural invasion were independent predictors for long-term survivors. Our results may help to plan and select post-operative adjuvant therapy for patients with PDAC who received NAT and pancreatoduodenectomy based on the pathologic data.

## 1. Introduction

Pancreatic ductal adenocarcinoma (PDAC) is a highly aggressive and lethal malignancy. It is the third leading cause of cancer-related deaths in the United States with a poor 5-year survival rate of approximately 11% [1]. The incidence of pancreatic cancer has increased worldwide and is projected to be the second-most-common cause of cancer-related deaths in the United States by 2030 [2,3]. Despite recent advancements in the field of oncology including immunotherapy and targeted therapy, the survival for patients with PDAC has only improved slightly over the past few decades [3]. Surgical resection continues to provide the only potential cure for PDAC. 

The neoadjuvant therapy (NAT) approach is more commonly used to treat patients with potentially resectable PDAC and is currently the standard of care for those with borderline resectable and high-risk resectable disease according to the current National Comprehensive Cancer Network (NCCN) and the American Society of Clinical Oncology (ASCO) guidelines [4]. For patients with locally advanced disease, NAT can help select those most suitable for resection. Several neoadjuvant chemotherapy regimens with or without radiation therapy have been used to treat patients with potentially resectable, borderline resectable, or locally advanced PDAC [5]. These regimens include gemcitabine-based regimens, such as gemcitabine monotherapy, gemcitabine plus capecitabine, nab-paclitaxel, or S1, and fluoropyrimidine-based regimens, such as folinic acid, 5-FU, irinotecan and oxaliplatin (FOLFIRINOX), and modified FOLFIRINOX [5,6,7,8]. A recent meta-analysis of 38 studies suggested that NAT may be offered as an acceptable standard of care for patients with potentially resectable PDAC [9].

Patients with PDAC often show heterogenous responses to NAT and variable clinical outcomes. Previous studies of post-therapy pancreatectomy specimens have shown that the majority of patients (>80%) demonstrated moderate or minimal responses to NAT, which is associated with poor survival. Only a minority (12.6–18.6%) of PDAC patients demonstrate a complete or near-complete response to NAT, which is associated with better survival [10,11,12,13]. In addition to tumor response grading (TRG), several other pathologic parameters, including tumor differentiation, pathologic primary tumor stage (ypT), lymph node stage (ypN), perineural invasion, vascular invasion, resection margins, tumor involvement of superior mesenteric vein/portal vein, and integrated pathologic score, have also been shown to affect the clinical outcomes and survival in PDAC patients who received NAT [10,13,14,15,16,17,18,19]. In our previous studies of PDAC patients who received NAT [10,13,14,15,16,17,18,19], we noticed that there were a significant number of patients who survived longer than 5 years (long-term survivors) and a smaller percentage of patients who survived less than 15 months (short-term survivors). However, the clinicopathologic parameters that are associated with long-term and short-term survivors in PDAC patients who received NAT have not been examined.

In this retrospective study, we systematically examined and compared clinicopathologic characteristics of short-term survivors (<15 months) and long-term survivors (>60 months) with the intermediate-term survivors (15–60 months) in a large cohort of 561 patients who received NAT and pancreatoduodenectomy (PD) for PDAC at our institution.

## 2. Materials and Methods

### 2.1. Patient Population

This study was approved by the institutional review board of the University of Texas MD Anderson Cancer Center. Our study population consisted of 561 PDAC patients (313 male and 248 female) who were diagnosed between January 1999 and December 2017, completed NAT, and underwent pancreatoduodenectomy at our institution. The pretreatment diagnosis of PDAC was confirmed in all cases by reviewing the biopsies and/or fine needle aspiration cytology. We excluded patients who had undergone distal pancreatectomy and those who had undergone pancreatoduodenectomy for other neoplasms. The median age for our study population was 64.2 years (range: 34.5–85.4 years). Tumor resectability prior to NAT was evaluated based on the computed tomographic (CT) scan optimized for pancreatic imaging as previously described [20].

### 2.2. Pathologic Evaluation

All pancreatoduodenectomy specimens were evaluated and reported using a standardized protocol at our institution, which include histologic type, differentiation/histologic grade, tumor location, tumor size, presence or absence of lymphovascular or perineural invasion, number of positive and total lymph nodes, TRG, tumor involvement of an extrapancreatic tissue/organ, and margin status [18,21]. The uncinate/superior mesenteric artery margin was considered as positive if the tumor extended to ≤1.0 mm to the inked margin. All other margins were routinely evaluated by en face section(s), and the margin was considered as positive if carcinoma was present. The ypTNM was grouped according to the American Joint committee on Cancer (AJCC) Cancer Staging Manual 8th edition [22]. Tumor response grading was evaluated using the MD Anderson (MDA) or the College of American Pathologists (CAP) grading systems. Given the small number of patients with ypT0 or MDA grade 0, we combined ypT0 and ypT1 as one group (ypT0/ypT1) and MDA grade 0 and 1 as one tumor response group for the multifactorial logistic analysis.

### 2.3. Clinical and Follow-Up Data

The demographic, clinical, pathologic, and follow-up data of our study population were retrieved from a prospectively maintained multidisciplinary pancreatic cancer database and verified by reviewing patients’ medical records and reviewing the U.S. Social Security Index when necessary. Overall survival was calculated from the date of first diagnosis to the date of death or the last follow-up date (if death did not occur). Based on the overall survival, we grouped our patients into three groups: the short-term survivors (overall survival < 15 months), the intermediate-term survivors (overall survival: 15–60 months), and the long-term survivors (overall survival > 60 months).

### 2.4. Statistical Analysis

Correlations between the survivor groups with different clinicopathologic parameters were performed using chi-square analyses or Fischer’s exact tests for categorical data. The independent-samples *t* test or one-way ANOVA was used to compare the means among different survivor groups for continuous variables. Multivariate logistic regression analyses were performed to identify the independent clinicopathologic parameters that are predictive of the short-term and long-term survivor groups. All statistical analyses were performed using Statistical Package for Social Sciences software for Windows (Version 26, SPSS, Inc., Chicago, IL, USA). A 2-sided significance level of <0.05 was used for all statistical analyses.

## 3. Results

### 3.1. Patient Characteristics

Among 561 patients, 386 (68.8%) were potential resectable, 136 (24.2%) were borderline resectable, and 39 (7.7%) had locally advanced disease. A total of 182 (32.4%) received neoadjuvant fluoropyrimidine-based chemotherapy with radiation (182), 328 (58.5%) received gemcitabine-based chemotherapy with radiation, 36 (6.4%) received gemcitabine-based chemotherapy, and 15 (2.7%) received FOLFIRINOX. Totals of 16 (2.9%), 194 (34.6%), 284 (50.6%), and 67 (11.9%) were ypT0, ypT1, ypT2, and ypT3, respectively, and 259 (46.2%), 194 (34.6%), and 108 (19.3%) were ypN0, ypN1, and ypN2, respectively. A total of 350 (62.4%) had a well to moderately differentiated histology and 211 (37.6%) had a poorly differentiated tumor. Totals of 16 (2.9%), 69 (12.3%), and 476 (84.8%) had MD Anderson grade 0, 1, and 2 responses, respectively, and 16 (2.9%), 69 (12.3%), 304 (54.2%), and 172 (30.6%) had CAP grade 0, 1, 2, and 3 responses, respectively. R0 (margin-negative microscopically) and R1 resection (margin-positive microscopically) were achieved in 483 (86.1%) and 78 (13.9%), respectively. The median overall survival was 35.1 months (range: 7.6 months to 257.5 months). There were 60 (10.7%) short-term survivors, 352 (62.7%) intermediate-term survivors, and 149 (26.6%) long-term survivors, respectively.

### 3.2. The Clinicopathologic Features of Short-Term Survivor Group

Comparisons of clinicopathologic parameters between the short-term survivor group and the intermediate-term survivor group are shown in Table 1. The short-term survivor group was associated with male gender (*p* = 0.03), poor differentiated histology (*p* = 0.006), more positive lymph nodes (*p* = 0.04), higher ypN stage (*p* = 0.002), and higher positive lymph node ratio (*p* = 0.03). Interestingly, compared to the intermediate-term survivor group, more patients in the short-term survival group were classified as potentially resectable before the initiation of NAT (83.3% vs. 66.8%, *p* =0.01). There were no significant differences between these two groups in age, NAT regimens, tumor size, ypT stage, total number of lymph nodes examined, lymphovascular and perineural invasion, margin status, tumor response grading using either the MDA or CAP grading system, and recurrence/metastasis (*p* > 0.05, Table 1). The mean duration of NAT was 3.0 ± 1.9 months for the short-term survivor group compared to 4.4 ± 2.7 months for the intermediate-term survivors (*p* < 0.001).

### 3.3. The Clinicopathologic Features of Long-Term Survivor Group

Comparisons of the clinicopathological parameters between the long-term survivor group and the intermediate-term survivor group are shown in Table 2. The long-term survivor group had smaller tumor size (*p* = 0.001), lower ypT stage (*p* = 0.001), fewer positive lymph nodes (*p* < 0.001), lower ypN stage (*p* < 0.001), lower positive lymph node ratio (*p* < 0.001), lower rate of lymphovascular invasion (*p* = 0.001) and perineural invasion (*p* < 0.001), better tumor response grading using either the MDA or CAP grading system (*p* < 0.001), and less frequent recurrence/metastasis (*p* < 0.001). There were no significant differences in age, gender, NAT regimens, tumor resectability prior to NAT, total number of lymph nodes examined, tumor differentiation, and margin status (*p* > 0.05). The mean duration of NAT was 4.1 ± 3.9 months for the long-term survivor group compared to 4.4 ± 2.7 months for the intermediate-term survivors (*p* = 0.28).

### 3.4. Comparison of the Clinicopathologic Features of Long-Term Survivor Group with Short-Term Survivor Group

**Figure 1:**
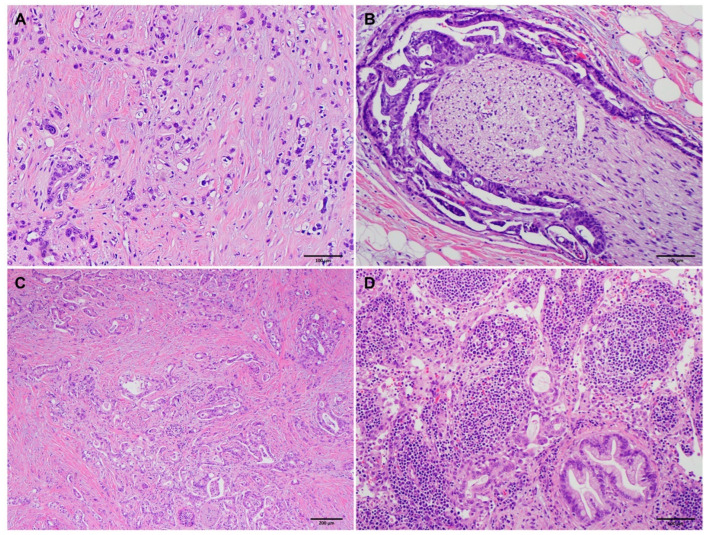
Representative micrographs showing poorly differentiated histology ((**A**), original magnification 100×), perineural invasion ((**B**), original magnification 100×), minimal response to neoadjuvant therapy ((**C**), original magnification 40×), and metastatic adenocarcinoma in a lymph node ((**D**), original magnification 100×) in four different short-term survivors.

**Figure 2:**
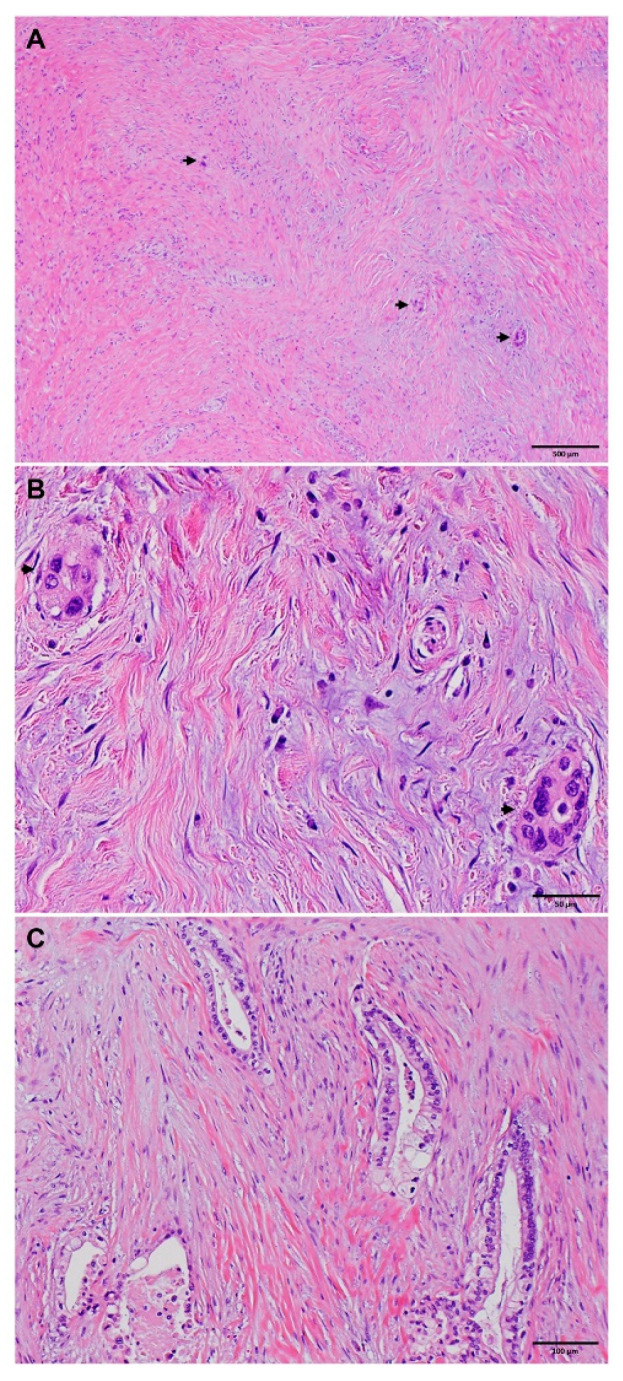
Representative micrographs showing near-complete response to neoadjuvant therapy ((**A**,**B**) in a long-term survivor). (**A**) Extensive fibrosis with microscopic foci of residual viable tumor (marked with arrows, original magnification 20×). (**B**) Higher magnification showing the microscopic foci of residual viable adenocarcinoma cells (marked with arrows, original magnification 200×). (**C**) Representative micrographs showing well-differentiated histology (original magnification 100×) in a long-term survivor.

Comparisons of clinicopathologic parameters between the long-term survivor group and the short-term survivor group are shown in Table 3. Compared to the short-term survivor group, the long-term survivor group was associated with younger age (*p* = 0.04) and female gender (*p* = 0.02), had smaller tumor size (*p* = 0.002), lower ypT stage (*p* = 0.04), better tumor differentiation (*p* = 0.001), lower rate of lymphovascular invasion (*p* < 0.001) and perineural invasion (*p* < 0.001), better tumor response grading using the MDA or CAP grading system (*p* = 0.04), fewer positive lymph nodes (*p* < 0.001), lower positive lymph node ratio (*p* < 0.001), lower ypN stage (*p* < 0.001), and less frequent recurrence/metastasis (*p* < 0.001). Representative histologic features that are associated with short- and long-term survivors are shown in Figure 1 and Figure 2. No significant differences in NAT regimens, tumor resectability prior to NAT, total number of lymph nodes examined, and margin status (*p* > 0.05) were found.

### 3.5. Multivariate Logistic Regression Analyses

Using the binary multivariate logistic regression analysis to predict short-term versus intermediate-term survivor groups, we found that ypN stage (*p* = 0.006) and tumor differentiation (*p* = 0.01) were independent factors that differentiated the two groups (Table 4 and Figure 1). Pretreatment tumor resectability (*p* = 0.07), TRG (*p* = 0.06), perineural invasion (*p* = 0.06), and gender (*p* = 0.15) were not statistically significant. On the other hand, the logistic regression analyses to differentiate long- vs. intermediate-term survivor groups discovered ypN stage (*p* = 0.003), tumor response grading (*p* = 0.04), and perineural invasion (*p* = 0.04) as independent factors that are associated with the long-term survivor group (Table 5). In particular, we found that better tumor response grading, lower ypN stage, and the absence of perineural invasion were associated with long- vs. intermediate-term survival (Figure 2). These logistic regression results present a consistent picture regarding the important clinicopathological parameters that were predictive of length of survival, which can be used clinically as prognostic markers.

## 4. Discussion

In this study, we examined clinicopathologic characteristics of short- and long-term survivor groups and compared them to an intermediate-term survivor group in a cohort of 561 patients with PDAC who underwent neoadjuvant therapy prior to pancreatoduodenectomy. We found that ypN stage and tumor differentiation were independent factors that were associated with the short-term survivor group. The independent factors that were associated with the long-term survivor group were ypN stage, tumor response grading, and perineural invasion. Our results highlight the importance of accurate pathologic evaluation in pancreatoduodenectomy specimens from PDAC patients who received neoadjuvant therapy.

The reported long-term survivor (survival ≥ 5 year) rate in patients with PDAC who received up-front surgical resection and adjuvant therapy ranges from 12.1% to 27.0% [23,24,25,26,27,28,29]. Consistent with previous studies, we showed that the long-term survivor rate was (26.6%) in PDAC patients who received NAT and pancreatoduodenectomy. However, the clinicopathologic parameters associated with long-term survivors of PDAC after NAT and pancreatoduodenectomy have not been studied in a large cohort of patients. In this study, we demonstrated that the long-term survivor group had a significantly lower ypN stage, fewer positive lymph nodes, lower positive lymph node ratio, smaller tumor size, lower ypT stage, lower rates of lymphovascular invasion and perineural invasion, better tumor response grading, and less frequent recurrence/metastasis compared to the intermediate-term survivor group. In multifactorial logistic regression analyses, ypN stage and perineural invasion were independent factors that are associated with the long-term survivor group. In addition, we showed for the first time that tumor response grading was associated with long-term survivors in PDAC patients who received NAT and pancreatoduodenectomy. Our results are consistent with previous studies showing that ypN stage and perineural invasion are associated with long-term survivors in PDAC patients who received up-front surgical resection [22,24,29,30,31,32]. Patient age and tumor differentiation were not associated with long-term survivors in this study.

Very few studies have examined the clinicopathologic parameters that are associated with short-term survivors for PDAC patients who underwent pancreatectomy. Takeuchi et al. examined the pre-operative predictors of short-term survivors of PDAC patients who underwent surgical resection. They found that the short-term survivors of PDAC patients (overall survival < 12 months) was negatively associated with patient age, tumor size of ≥3 cm based on a computerized tomography (CT) scan, and the level of serum carcinoembryonic antigen (CEA) [33]. A meta-analysis by Agalianos et al. showed that positive para-aortic lymph nodes following pancreatectomy was associated with a lower one-year survival rate [34]. In our cohort of patients with PDAC who received NAT and pancreatoduodenectomy, only 24 of 561 patients (4.3%) survived < 12 months. To increase the number of short-term survivors and the representation of this study group, we used the overall survival time of 15 months as the cut off for the short-term survivor group and identified 60 (10.7%) short-term survivors in this study. We demonstrated that male gender, poor differentiated histology, higher number of positive lymph nodes, higher ypN stage, and higher positive lymph node ratio were associated the short-term survivor group compared to the intermediate-term survivor group. In multivariate logistic regression analyses, ypN stage and tumor differentiation were independent predictors for short-term survivors. We did not observe a significant association between the short-term survivor group and age, tumor size, ypT stage, lymphovascular invasion, or margin status.

Accurate lymph node staging plays a critical role in patient prognosis in both PDAC patients who underwent upfront surgical resection and those who received NAT followed by resection with curative intent [13,35]. In this study, we found that ypN stage was an independent factor associated with both short-term and long-term survivor groups by multivariate logistic regression analyses. This result is consistent with the findings of our previous studies showing that the number of positive lymph nodes, ypN stage, and the positive lymph node ratio are independent prognostic factors for both disease free survival (DFS) and overall survival (OS) in patients with PDAC who received NAT and pancreatectomy [13,16,18,21].

A recent study by Tong et al. showed that patients with PDAC who received neoadjuvant FOLFIRINOX were younger and had higher rates of borderline resectable and locally advanced disease, but had higher rates of complete or near-complete pathologic responses and lower ypN stage compared to those who received neoadjuvant gemcitabine/nab-paclitaxel [6]. They also showed that FOLFIRINOX plus radiation was associated with decreased lymph node metastasis and lower ypN stage than those who received FOLFIRINOX alone. However, they did not observe a significant difference in survival between the FOLFIRINOX and gemcitabine/nab-paclitaxel groups [6]. Similar results were also reported from a Phase II study, which compared neoadjuvant FOLFIRINOX versus gemcitabine/nab-paclitaxel for borderline-resectable PDAC (NUPAT-01) and showed no significant difference in the 3-year survival between these two regimens. [36] Consistent with these results, we did not find a significant association of different NAT regimens with either short-term or long-term survivors. It should be noted that the majority (90.9%) of PDAC patients in this study received neoadjuvant fluoropyrimidine-based or gemcitabine-based chemotherapy with radiation, only 6.4% received gemcitabine-based chemotherapy alone, and 2.7% received FOLFIRINOX alone. Prospective randomized clinical trials are needed to compare the efficacy of different NAT regimens with or without radiation.

Limitations of this study include that this is a single-institution retrospective study at a tertiary cancer center, which may have potential bias in the patient population. In addition, our study population does not include any patients who underwent neoadjuvant therapy but did not undergo surgical resection, due to tumor progression during treatment. Another limitation of this study is that our patient population spanned a period of 18 years between 1999 and 2017, and neoadjuvant regimens have evolved over this time period. Therefore, our study results should be interpreted cautiously. Despite the stated limitations, our study is the first large study to examine clinicopathologic characteristics of short-term, intermediate-term, or long-term survivors of PDAC patients treated with neoadjuvant therapy followed by pancreatoduodenectomy.

## 5. Conclusions

In summary, our study demonstrates that ypN stage is an independent predictor of both short- and long-term survivors in patients with PDAC who received NAT and pancreatoduodenectomy. In addition, we demonstrated that tumor differentiation is an important predictor of short-term survivors and that tumor response grading and perineural invasion are important predictors of long-term survivors. Our results may help to select and plan post-operative adjuvant therapy for patients with PDAC who received NAT and pancreatoduodenectomy based on the pathologic data.

## Figures and Tables

**Table 1:** Comparison of the clinicopathologic factors between short-term and intermediate-term survivors.

Characteristics	Short-Term	Intermediate-Term	*p* Value
Mean age ± SD (years)	66.0 ± 9.8	63.5 ± 9.3	0.06
Sex			0.03
Female	18	159	
Male	42	193	
Neoadjuvant therapy			0.214
FP-based chemo + radiation	16	119	
Gem-based chemo + radiation	41	193	
Gem-based chemo	2	30	
FOLFIRINOX	1	10	
Resectability			0.04
Potentially resectable	50	235	
Borderline resectable	8	92	
Locally advanced	2	25	
Differentiation			0.006
Well-moderate	26	221	
Poor	34	131	
Mean tumor size ± SD (cm)	2.9 ± 1.2	2.7 ± 1.3	0.13
ypT stage			0.36
ypT0/ypT1	21	115	
ypT2	28	193	
ypT3	11	44	
Lymphovascular invasion			0.12
Negative	22	169	
Positive	38	183	
Perineural invasion			0.052
Negative	6	73	
Positive	54	279	
ypN stage			0.002
ypN0	12	153	
ypN1	31	119	
ypN2	17	80	
Mean number of positive nodes ± SD	3.1 ± 3.7	2.1 ± 3.3	0.04
Mean total number of nodes ± SD	23.8 ± 9.6	25.9 ± 10.3	0.14
Mean positive lymph node ratio ± SD	0.12 ± 0.13	0.09 ± 0.12	0.03
MDA Tumor Response Grade			0.46
MDA grade 0	0	7	
MDA grade 1	7	32	
MDA grade 2	53	313	
CAP Tumor Response Grade			0.50
Grade 0	0	7	
Grade 1	7	32	
Grade 2	31	203	
Grade 3	22	110	
Margin status			0.70
Negative	52	296	
Positive	8	56	
Recurrence/metastasis			0.15
None	8	63	
Local	8	77	
Distant	44	212	

Abbreviations: SD, standard deviation; FP, fluoropyrimidine; Gem, gemcitabine; chemo, chemotherapy.

**Table 2:** Comparison of the clinicopathologic factors between long-term and intermediate-term survivors.

	Long-Term	Intermediate-Term	*p* Value
Mean age ± SD (years)	63.0 ± 9.6	63.5 ± 9.3	0.59
Sex			0.63
Female	71	159	
Male	78	193	
Neoadjuvant therapy			0.08
FP-based chemo + radiation	47	119	
Gem-based chemo + radiation	94	193	
Gem-based chemo	4	30	
FOLFIRINOX	4	10	
Resectability			0.86
Potentially resectable	101	235	
Borderline resectable	36	92	
Locally advanced	12	25	
Differentiation			0.19
Well-moderate	103	221	
Poor	46	131	
Mean tumor size ± SD (cm)	2.24 ± 1.52	2.67 ± 1.27	0.001
ypT stage			0.001
ypT0/ypT1	74	115	
ypT2	63	193	
ypT3	12	44	
Lymphovascular invasion			0.001
Negative	96	169	
Positive	53	183	
Perineural invasion			<0.001
Negative	59	73	
Positive	90	279	
ypN stage			<0.001
ypN0	94	153	
ypN1	44	119	
ypN2	11	80	
Mean number of positive nodes ± SD	1.04 ± 2.53	2.14 ± 3.30	<0.001
Mean total number of nodes ± SD	25.58 ± 10.49	25.88 ± 10.29	0.77
Mean positive lymph node ratio ± SD	0.036 ± 0.068	0.087 ± 0.124	<0.001
MDA Tumor Response Grade			<0.001
MDA grade 0	9	7	
MDA grade 1	30	32	
MDA grade 2	110	313	
CAP Tumor Response Grade			<0.001
Grade 0	9	7	
Grade 1	30	32	
Grade 2	70	203	
Grade 3	40	110	
Margin status			0.07
Negative	135	296	
Positive	14	56	
Recurrence/metastasis			<0.001
None	97	63	
Local	13	77	
Distant	39	212	

Abbreviations: SD, standard deviation; FP, fluoropyrimidine; Gem, gemcitabine; chemo, chemotherapy.

**Table 3:** Comparison of the clinicopathologic factors between short-term and long-term survivors.

Characteristics	Short-Term	Long-Term	*p* Value
Mean age ± SD (years)	66.0 ± 9.8	63.0 ± 9.6	0.04
Sex			0.02
Female	18	71	
Male	42	78	
Neoadjuvant therapy			0.86
FP-based chemo + radiation	16	47	
Gem-based chemo + radiation	41	94	
Gem-based chemo	2	4	
FOLFIRINOX	1	4	
Resectability			0.07
Potentially resectable	50	101	
Borderline resectable	8	36	
Locally advanced	2	12	
Differentiation			0.001
Well-moderate	26	103	
Poor	34	46	
Mean tumor size ± SD (cm)	2.93 ± 1.23	2.24 ± 1.52	0.002
ypT stage			0.04
ypT0/ypT1	21	74	
ypT2	28	63	
ypT3	11	12	
Lymphovascular invasion			<0.001
Negative	22	96	
Positive	38	53	
Perineural invasion			<0.001
Negative	6	59	
Positive	54	90	
ypN stage			<0.001
ypN0	12	94	
ypN1	31	44	
ypN2	17	11	
Mean number of positive nodes ± SD	3.12 ± 3.65	1.04 ± 2.53	<0.001
Mean total number of nodes ± SD	23.75 ± 9.62	25.58 ± 10.49	0.24
Mean positive lymph node ratio ± SD	0.124 ± 0.134	0.036 ± 0.068	<0.001
MDA Tumor Response Grade			0.04
MDA grade 0	0	9	
MDA grade 1	7	30	
MDA grade 2	53	110	
CAP Tumor Response Grade			0.07
Grade 0	0	9	
Grade 1	7	30	
Grade 2	31	70	
Grade 3	22	40	
Margin status			0.46
Negative	52	135	
Positive	8	14	
Recurrence			<0.001
None	8	97	
Local	8	13	
Distant	44	39	

Abbreviations: SD, standard deviation; FP, fluoropyrimidine; Gem, gemcitabine; chemo, chemotherapy.

**Table 4:** Multivariate logistic regression analysis of short-term vs. intermediate-term survivors.

Characteristics	Number of Patients	OR (95% CI)	*p* Value
Sex			
Female	177	1.00	
Male	235	1.57 (0.85–2.90)	0.15
Resectability			0.07
Potentially resectable	285	1.00	
Borderline resectable	100	3.22 (0.72–14.49)	0.13
Locally advanced	27	1.48 (0.29–7.61)	0.64
Tumor differentiation			
Well-moderate	247	1.00	
Poor	165	2.09 (1.18–3.72)	0.01
ypN stage			0.006
ypN0	165	1.00	
ypN1	150	3.33 (1.59–6.97)	0.001
ypN2	97	2.67 (1.15–6.18)	0.02
Tumor response grading			
MDA grade 0 or 1	46	1.00	
MDA grade 2	366	3.02 (0.97–9.45)	0.06
Perineural Invasion			
Negative	79	1.00	
Present	333	2.87 (0.95–8.64)	0.06

Abbreviations: OR, Odds ratio; CI, confidence interval.

**Table 5:** Multivariate logistic regression analysis of long-term vs. intermediate-term survivors.

Characteristics	Number of Patients	OR (95% CI)	*p* Value
Neoadjuvant therapy			0.41
FP-based chemo + radiation	166	1.00	
Gem-based chemo + radiation	287	1.21 (0.78–1.86)	0.40
Gem-based chemo	34	0.50 (0.16–1.53)	0.22
FOLFIRINOX	14	1.02 (0.29–3.62)	0.97
ypT stage			0.44
ypT0/ypT1	189	1.00	
ypT2	256	0.76 (0.48–1.20)	0.24
ypT3	56	0.71 (0.33–1.51)	0.37
ypN stage			0.003
ypN0	247	1.00	
ypN1	163	0.72 (0.46–1.12)	0.14
ypN2	91	0.30 (0.15–0.60)	0.001
Tumor Response Grading			
MDA grade 0 or 1	78	1.00	
MDA grade 2	423	0.55 (0.32–0.97)	0.04
Lymphovascular invasion			
Negative	265	1.00	
Positive	236	0.86 (0.54–1.37)	0.52
Perineural invasion			
Negative	132	1.00	
Positive	369	0.61 (0.38–0.98)	0.04
Margin			
Negative	431	1.00	
Positive	70	0.92 (0.47–1.81)	0.82

Abbreviations: FP, fluoropyrimidine; Gem, gemcitabine; chemo, chemotherapy; OR, Odds ratio; CI, confidence interval.

## Data Availability

The data are unavailable due to privacy or ethical restrictions.
